# A Pott’s Puffy Tumor Associated with Epidural - Cutaneous Fistula and Epidural Abscess: Case Report

**DOI:** 10.4274/balkanmedj.2016.1304

**Published:** 2017-05-15

**Authors:** Aleksandar Perić, Milanko Milojević, Dražen Ivetić

**Affiliations:** 1 Department of Otorhinolaryngology, Military Medical Academy School of Medicine, Belgrade, Serbia; 2 Department of Neurosurgery, Military Medical Academy School of Medicine, Belgrade, Serbia

**Keywords:** Pott’s puffy tumour, frontal sinusitis, osteomyelitis, antibacterial agents, nasal surgical procedures

## Abstract

**Background:**

Pott’s puffy tumour is characterized by a fluctuate swelling of the frontal region as a result of osteomyelitis of the frontal bone. This inflammatory lesion may propagate endocranially, resulting in acute meningitis, epidural abscess, subdural empyema, cavernous sinus trombophlebitis, cerebritis, and frontal lobe abscess of the brain.

**Case Report:**

We present an unusual case of a 33-year-old man suffering from Pott’s puffy tumour whose condition was further complicated by a draining epidural-cutaneous fistula and an epidural abscess. We confirmed the diagnosis by contrast-enhanced computed tomography scanning and magnetic resonance imaging of the head. After intense antibiotic treatment, we performed a combined endoscopic and external surgical approach with drainage of abscesses, evacuation of pus and bone sequestrate and excision of fistulous lesion. The treatment was prolonged with four weeks’ antibiotic administration.

**Conclusion:**

Subperiosteal abscess of the frontal bone is an extremely rare complication of frontal sinusitis. This lesion may propagate endocranially, resulting in dangerous intracranial inflammatory lesions. Early diagnosis, medication and surgical therapy are very important in reducing morbidity and mortality.

Pott’s puffy tumour (PPT), originally described by Sir Percival Pott, surgeon from the London, in 1760, is diagnosed as a subperiosteal abscess of the anterior wall of the frontal sinus originating from osteomyelitis of the frontal bone (1). This condition results from acute or chronic inflammation of the frontal sinus, although trauma and previous surgical procedures have been proposed (2). Although this complication mainly affects adolescents, it can be found in all ages (1,2). In this report, we describe the case of a patient with PPT complicated with epidural-cutaneous fistula and epidural abscess, treated by a combined endoscopic and external surgical approach. To our knowledge, only one similar case has been previously described in the world literature (3).

## CASE PRESENTATION

**Figure 1:**
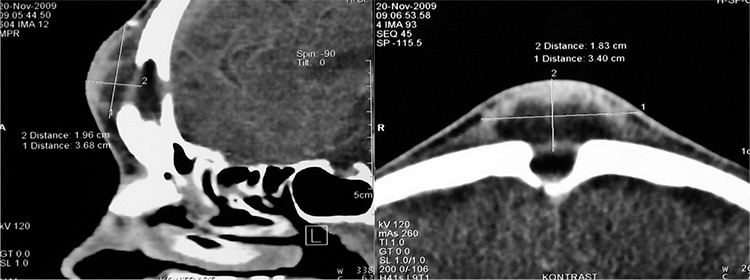
Sagittal (a) and axial (b) contrast-enhanced CT scan of the paranasal sinuses show osteomyelitis of the frontal bone with destruction of the anterior and posterior frontal sinus wall. Note the presence of fistulous communication between the subperiosteal and epidural abscess.

**Figure 2:**
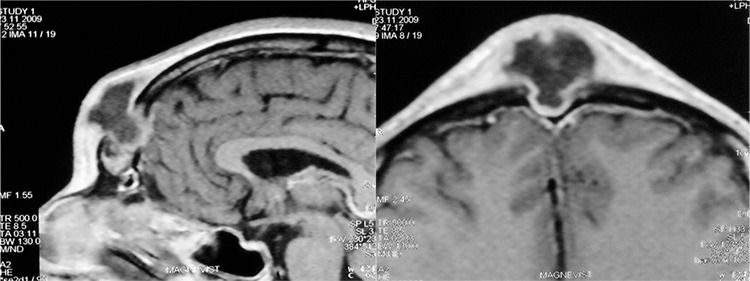
The T2-weighted sagittal (a) and axial (b) MRI images show frontal Pott’s puffy tumour with epidural-cutaneous fistula. Note the absence of other intracranial complications.

A 33-year-old male patient presented with headache of the frontal region for the last 3 years, midline fluctuate swelling of the forehead and recurrent foul-smelling purulent discharge from a sinus over the forehead for the last 5 months. He also complained of nasal obstruction and bilateral intermittent nasal mucopurulent secretion. The patient had no traumas or previous surgical intervention in the frontal region. He was febrile, with a temperature up to 38.5 °C for the previous 7 days. Nasal endoscopy revealed left side middle turbinate hypertrophy, septal deviation to the right side and bilateral middle meatus mucopurulent discharge. Laboratory tests yielded an erytrocyte sedimentation rate of 78 mm/h and white blood cell count of 13600x10^9^/L. A contrast-enhanced computed tomograpy (CT) scanning with coronal, sagittal and axial reconstructions was performed. The CT scan showed the presence of an osteomyelitis of the frontal bone with destruction of the anterior and posterior frontal sinus wall. A subperiosteal (3.60x1.96 cm) and an epidural abscess that were connected to each other through a fistulous canal were seen. CT scan also showed bilateral extensive opacification of the anterior ethmoid cells and frontal recess and an extensive pneumatization of the left middle turbinate (Figure 1a, 1b). After intravenous gadolinium injection, a magnetic resonance imaging [magnetic resonance imaging (MRI), 1.5 Tesla] was performed. The examination confirmed the presence of osteomyelitis of the frontal bone with both abscesses and an epidural-cutaneous fistula (Figure 2a, 2b). No other intracranial complication was found.

The bacterial cultures taken from the frontal fistulous canal grew *Staphylococcus aureus* and an anaerobe Peptostreptococus prevoti, and from the nasal cavity *Streptococcus pneumoniae*, *Haemophilus influenzae* and *Staphylococcus aureus*. Three intravenous antibiotics [cephtriaxone (Longaceph^®^, Galenika AD, Belgrade, Serbia) 2.0 g, two times daily; metronidazole (Orvagyl®, Galenika AD, Belgrade, Serbia) 0.5 g, three times daily; amoxicillin-clavulanate (Augmentin^®^, Glaxo Smith Kline, Uxbridge, Middlesex, United Kingdom) 1.2 g, three times daily] were administered for 2 weeks to control the active disease. Two days after the start of antibiotic therapy, the patient was afebrile and without secretion from the fistula. Surgical treatment included a combined endoscopic and external approach. The patient underwent bilateral endoscopic anterior ethmoidectomy and left-sided lateral resection of the concha bullosa under general anaesthesia. The surgery was continued by external approach performed by a neurosurgeon and rhinologist. We performed a craniotomy using the bicoronal approach. After the surgical drainage of the epidural abscess and debridement of the osteomyelitic focus, the fistulous canal and inflamed mucosa of the frontal sinus were removed. The granulation tissue overlying the dura mater was carefully excised. No defect of on the dura was found. At the end, bony defects were successfully reconstructed using a temporal muscle flap. Histopathological examination of the excised tissue showed chronic non-specific granulation with bony sequestration and foreign body giant cell reaction. The treatment was continued with 2 weeks of intravenous and 4 weeks of oral antibiotics. The patient’s post-operative course was uneventful. He had a complete recovery and after 7 years of follow-up, there has been no recurrence.

## DISCUSSION

PPT is very rare complication of frontal sinusitis in the age of antibiotics. It is found mainly in adolescents. According to the world English-language literature, only 30-40 cases of adult patients with PPT were reported between 1990 and 2016 (4,5). PPT is a risk factor for endocranial complications (epidural abscess, subdural abscess, acute meningitis and frontal lobe abscess), which have been found in about 60-85% of patients with PPT (5). Anatomical characteristics of frontal sinus and its specific veins could be the factors influencing the development of PPT and its complications. Pneumatization of the frontal sinus starts at about one year old and continues until 18 years of age (6). Many anatomical factors may narrow the frontal recess until complete obstruction. These include overpneumatized ethmoid bulla, extensivelly pneumatized middle turbinate, enlargement of agger nasi cells, among others. Sinus obstruction by anatomical anomalies and inflamed mucosa leads to ciliary dysfunction and mucus stagnation that results in an anaerobic environment in the sinus (6). In the case of our patient, an overpneumatized middle turbinate affected the frontal recess drainage on the left side. Therefore, endoscopic sinus surgery (ESS) with removal of these anatomical variations should be an important part in the treatment of PPT patients. Because the veins of the frontal sinus mucosa communicate through the diploic veins with the periosteum veins and with the dural venous plexus, septic trombi can potentially migrate from the frontal sinus and propagate to the periosteum and to the dura (5). However, according to some authors, atypical forms of PPT without underlying osteomyelitis are becoming more common (7). Progresive trombophlebitis without bone inflammation is also a potential cause of PPT and its intracranial complications. Infectious trombophlebitis can extend posteriorly, causing epidural abscess, meningitis or frontal lobe abscess (7).

Intranasal cocaine abuse is also an important cause of PPT. Inhaled cocaine compromises local blood circulation and, potentially, can destroy bony structures of the nose/paranasal sinuses (8). The several chronic diseases that affect the innate immune response, such as diabetes, chronic renal failure and aplastic anaemia could be risk factors for the development of PPT (4,5). The relatively frequent appearance of PPT in adolescents could be related to the continued growth of the frontal bone and the increased diploic vein flow in this period (5,9).

Early diagnosis of PPT is based on rhinological findings and radiological imaging. This rare but grave clinical entity can be misdiagnosed with benign or malignant neoplasms, soft-tissue and skin infections, and infected haematoma of the frontal area. Contrast-enhanced CT is the most adequate imaging method for the diagnosis of PPT (10). CT is the best method for the visualization of bony structures. It provides excellent visualization of air-bone and air-soft tissue interfaces. MRI provides a superior soft tissue resolution and more detail in the description of the extent of the disease, and enables better evaluation of the underlying subdural space and brain (5,10).

Osteomyelitis of the frontal bone, commonly associated with intracranial complications, requires swift management. Antibiotic therapy is of crucial importance. It is necessary to deal with *Staphylococcus aureus*, the most involved bacteria in the nasal/paranasal chronic inflammatory process. Therefore, antibiotics must be used to cover streptococci and anaerobes. Finally, the antimicrobial treatment should be adapted to the results of bacteriological analysis (4,6). It is necessary that selected antibiotics cross the blood-brain barrier and cover both aerobic and anaerobic flora. According to previous studies, the ideal combination is penicillin or vancomycin, third-generation cephalosporin and metronidazole (4,6). The use of antibiotics should be extended to at least 4-8 weeks after the surgery (4,5). Surgical treatment options include ESS and/or an external approach, including craniotomy, with the opening of the affected frontal sinus and radical debridement of the osteomyelitis process. Careful surgical intervention, with drainage of intracranial lesions and debridement of infected bone, is the keystone to successful treatment (4).
